# Supplementary material for: Susceptibility of Aedes aegypti Larvae to Temephos and Fenitrothion in Niamey (Niger) and Ouagadougou (Burkina Faso), Two West African Cities Recently Affected by Dengue
Source: Insects. 2025 Aug 22;16(9):870. doi: 10.3390/insects16090870 (PMC12471058; doi:10.3390/insects16090870)
Supplement: Supplementary file 1 [file insects-16-00870-s001.zip › insects-3703988-supplementary/insects 3703988-S1 table. 24 h mortality of Ae. aegypti larvae exposed to temephos and fenirothion. Niger.pdf]

Table S1. Twenty-four h mortality of *Ae. aegypti* larvae from Niamey exposed to temephos and fenirothion.

Insecticide	Strain	Dose (mg/L)	Total	Dead
Temephos	Niamey RD	0.0000	150	0
Temephos	Niamey RD	0.00441	151	21
Temephos	Niamey RD	0.00738	154	40
Temephos	Niamey RD	0.01038	161	59
Temephos	Niamey RD	0.01479	148	93
Temephos	Niamey RD	0.02217	145	126
Temephos	Niamey RD	0.03	156	146
Temephos	Niamey RG	0.0000	150	0
Temephos	Niamey RG	0.00441	119	35
Temephos	Niamey RG	0.00738	155	52
Temephos	Niamey RG	0.01038	154	84
Temephos	Niamey RG	0.01479	151	110
Temephos	Niamey RG	0.02217	157	136
Temephos	Niamey RG	0.03	155	141
Fenitrothion	Niamey RD	0.0000	150	0
Fenitrothion	Niamey RD	0.00441	151	1
Fenitrothion	Niamey RD	0.00591	142	20
Fenitrothion	Niamey RD	0.00738	151	31
Fenitrothion	Niamey RD	0.01107	148	68
Fenitrothion	Niamey RD	0.01479	149	111
Fenitrothion	Niamey RD	0.02217	154	146
Fenitrothion	Niamey RG	0.0000	150	0
Fenitrothion	Niamey RG	0.00441	150	15
Fenitrothion	Niamey RG	0.00591	144	55
Fenitrothion	Niamey RG	0.00738	146	62
Fenitrothion	Niamey RG	0.01107	146	113
Fenitrothion	Niamey RG	0.01479	151	143
Fenitrothion	Niamey RG	0.02217	153	152
