# Supplementary material for: Susceptibility of Aedes aegypti Larvae to Temephos and Fenitrothion in Niamey (Niger) and Ouagadougou (Burkina Faso), Two West African Cities Recently Affected by Dengue
Source: Insects. 2025 Aug 22;16(9):870. doi: 10.3390/insects16090870 (PMC12471058; doi:10.3390/insects16090870)
Supplement: Supplementary file 1 [file insects-16-00870-s001.zip › insects-3703988-supplementary/insects 3703988-S2 table. 24 h mortality of Ae. aegypti larvae exposed to temephos and fenitrothion. Burkina Faso.pdf]

Table S2. Dead and total tested number of *Aedes aegypti* Larvae from different breeding container types, after 24-Hour exposure to temephos and fenitrothion in Zongo, Ouagadougou.

Insecticide	Population	Dose (mg/L)	Total Tested	Dead
Temephos	Zongo drum	0.000	360	0
		0.004	255	108
		0.007	263	156
		0.010	262	173
		0.015	258	210
		0.022	262	241
		0.030	265	258
Fenitrothion	Zongo drum	0.000	398	0
		0.004	260	89
		0.006	264	139
		0.007	262	181
		0.011	258	218
		0.015	261	244
		0.022	265	264
Temephos	Zongo Plastic	0.000	267	0
		0.004	180	69
		0.007	179	88
		0.010	178	109
		0.015	175	135
		0.022	177	162
		0.030	175	163
Fenitrothion	Zongo Plastic	0.000	267	0
		0.004	179	56
		0.006	178	93
		0.007	177	117
		0.011	176	163
		0.015	179	176
		0.022	178	176
Temephos	Zongo metallic	0.000	139	0
		0.004	95	5
		0.007	86	46
		0.010	89	62
		0.015	94	78
		0.022	87	82
		0.030	87	83
Fenitrothion	Zongo metallic	0.000	135	0
		0.004	94	35
		0.006	85	44
		0.007	88	54
		0.011	84	63
		0.015	88	81
		0.022	89	88
